# Development of allele-specific multiplex PCR to determine the length of poly-T in intron 8 of *CFTR*

**DOI:** 10.7717/peerj.468

**Published:** 2014-07-08

**Authors:** Neng Chen, Anne E. Prada

**Affiliations:** 1 Department of Pathology and Laboratory Medicine, Beaumont Health System, Royal Oak, MI, USA; 2 Department of Pathology and Laboratory Medicine, Oakland University William Beaumont School of Medicine, Royal Oak, MI, USA

**Keywords:** Poly-T analysis, CFTR, Multiplex allele-specific PCR

## Abstract

Cystic fibrosis transmembrane conductance regulator (*CFTR*) gene mutation analysis has been implemented for Cystic Fibrosis (CF) carrier screening, and molecular diagnosis of CF and congenital bilateral absence of the vas deferens (CBAVD). Although poly-T allele analysis in intron 8 of *CFTR* is required when a patient is positive for R117H, it is not recommended for routine carrier screening. Therefore, commercial kits for *CFTR* mutation analysis were designed either to mask the poly-T allele results, unless a patient is R117H positive, or to have the poly-T analysis as a standalone reflex test using the same commercial platform. There are other standalone assays developed to detect poly-T alleles, such as heteroduplex analysis, High Resolution Melting (HRM) curve analysis, allele-specific PCR (AS-PCR) and Sanger sequencing. In this report, we developed a simple and easy-to-implement multiplex AS-PCR assay using unlabeled standard length primers, which can be used as a reflex or standalone test for *CFTR* poly-T track analysis. Out of 115 human gDNA samples tested, results from our new AS-PCR matched to the previous known poly-T results or results from Sanger sequencing.

## Introduction

Cystic fibrosis (CF) is one of the most common autosomal recessive disorders among northern European descendants. It affects lung, pancreas, liver, intestine and male reproductive systems. Diagnosis of CF is based on clinical manifestations plus sweat chloride tests, transepithelial nasal potential difference (NPD) measurements, or mutation analysis in cystic fibrosis transmembrane conductance regulator (*CFTR*) gene (Moskowitz et al., 2008). Molecular diagnosis of CF is indicated if a patient has two disease-causing mutations in *CFTR* (i.e., neither of the *CFTR* alleles function normally). *CFTR* mutations are also found in infertile males with congenital bilateral absence of the vas deferens (CBAVD). About 80% of men with CBAVD have at least one *CFTR* mutation (Mak et al., 1999). Therefore, molecular analysis of *CFTR* gene has become part of the routine for diagnosis of CF and/or CBAVD.

Multiple commercial products have been developed for *CFTR* testing (Johnson, Yoshitomi & Richards, 2007). All include the 23 mutations recommended by American College of Medical Genetics and Genomics (ACMG) and American Congress of Obstetricians and Gynecologists (ACOG) (Grody et al., 2001; Watson et al., 2004) for carrier screening. One of the criteria to select the mutations for the recommended *CFTR* mutation panel was that allele frequency of a mutation must be at least 0.1% in the general US population. Inevitably, the selected 23 mutations would have higher detection rate in non-Hispanic white and Ashkenazi Jewish population, who have the highest CF prevalence in general populations. The same recommended panel would lead to lower detection rate in other ethnic groups such as Hispanics, African Americans and Asians. For this reason, mutations with relatively high allele frequency in other minority ethnic groups (Macek et al., 1997; Rohlfs et al., 2011; Sugarman et al., 2004) were added in each panel to increase the detection rate. Therefore, each panel contained a varying number of mutations ranging from 30 to more than 100. Additional testing of the poly-Thymidine (poly-T) tract in intron 8 of *CFTR* is indicated when a patient is positive for R117H, one of the 23 recommended mutations (Grody et al., 2001). Poly-T tract has three alleles, 5, 7, and 9 Thymidine (T) in general population, with allele frequency of about 5%, 85% and 10%, respectively (Chillon et al., 1995). However, only one of the poly-T alleles, 5T, has reduced splicing of exon 9 and decreased expression of the full-length gene products (Chu et al., 1993). 5T alone is not considered a disease-causing CF mutation, but it is related to CBAVD (Chillon et al., 1995). Presence of both R117H and 5T allele on the same chromosome (i.e., R117H and 5T in *cis*-configuration) constitutes a disease-causing CF mutation (Bienvenu et al., 1993; Chmiel et al., 1999; Dean et al., 1990). As a result, poly-T tract testing was considered as a follow-up test (i.e., reflex test) and is included in almost all the commercial assays. Testing parents of patients is able to define whether R117H and 5T are from the one parent (i.e., in *cis*-configuration) or different parent (i.e., in *trans*-configuration), if 5T is detected in R117H positive samples. Because R117H and poly-T alleles are about 18 kb apart, long-range PCR has also been developed to determine the *cis*- or *trans*-relationship of R117H and 5T alleles to circumvent testing unaffected parents (Chen & Schrijver, 2011; Pont-Kingdon et al., 2004).

However, detection of sequence variations in the poly-T region of intron 8 (i.e., 5T, 7T or 9T) is not recommended for routine carrier screening. For this reason, some commercial assays mask the results of the poly-T tract and only provide poly-T results when the samples are R117H positive. Others have the poly-T assay in a separate reaction from the rest of the 23 mutations to serve as a standalone reflex test. When a clinician wants to test the poly-T tract for a male patient with clinical indication of CBAVD, a standalone poly-T test (i.e., poly-T test in a separate tube) would be ordered, because it is impossible to obtain the poly-T tract results with R117H negative samples if laboratories only run commercial *CFTR* mutation analysis with masked poly-T results. Running a standalone poly-T test on another platform could be cumbersome if the platform is not readily available in these laboratories. Moreover, with the recent advancement of next generation sequencing (NGS) technology, laboratories started to develop their own NGS*CFTR* testing. Alignment of the sequence reads in the poly-T region is extremely challenging as there are three common poly-T alleles and variable numbers of TG repeats at the 5′ of the poly-T tract in each individual allele. Commonly available NGS commercial software cannot align the region properly. End users have to write their own script in order to have the correct alignment in the poly-T region (Trujillano et al., 2013). This would be an issue for laboratories who do not have this type of bioinformatics support. In this case, a standalone poly-T assay can be implemented as part of *CFTR* molecular testing when NGS is applied to detect other mutations in the gene.

There are standalone poly-T assays developed for clinical research or testing, such as heteroduplex (Chillon et al., 1995), High Resolution Melting (HRM) curve analysis (Millson et al., 2005), and Allele-Specific PCR (AS-PCR) (Costa et al., 2008; Friedman et al., 1997). In addition, direct Sanger sequencing was also used to identify the variable length of the poly-T track (Chen & Schrijver, 2011; Chiang et al., 2009). The heteroduplex analysis involves running the nested PCR products on non-denaturing polyacrylamide gel to visualize the different number of thymidine in the poly-T track. HRM analysis is much easier to set up. After PCR reaction, collection of the melt curve signals is performed directly using the same tube without any treatment. However, patterns of the HRM curves for each individual sample could be different and complex, depending on combination of the poly-T with the adjacent TG repeats. This complicates the interpretation of the HRM results.

In contrast, interpretation of AS-PCR results is simpler. Presence or absence of the AS-PCR products would indicate the specific variant that is intended to be detected by the allele specific primer. Non-fluorescent AS-PCR was developed by Friedman et al. (1997) to detect 5T, 7T and 9T in the poly-T track. Three individual AS-PCR reactions, 5T, 7T and 9T were set up in three separate tubes for a sample. Following PCR, the presence and absence of the AS-PCR products were visualized on agarose gels, which are commonly used in molecular laboratories.

Despite its simplicity, it would be ideal if the reaction and detection could be done in a single tube for each sample. To avoid setting up three separate reactions for each poly-T allele, fluorescent labeling of each allele-specific primer in a different color made visualization of each poly-T allele in a single tube reaction possible (Costa et al., 2008). As it is necessary to run the final products on a fragment analysis instrument, such as capillary electrophoresis, the fluorescent labeling method may not be readily adaptable in certain laboratories. Although Sanger sequencing is the gold standard for mutation detection, it also requires capillary electrophoresis as is with labeled AS-PCR. Elucigene developed a multiplex AS-PCR product, CF Poly-T (Cat. Code: PT003B2 ), which was able to distinguish each poly-T allele on agarose gels by size (Elucigene Diagnostics, 2004). The concept was to add different lengths of 5′ overhang, ranging from 0 to 64 bases for each allele-specific primer (Iain et al., 2001). This leads to a product size of 105 bp, 132 bp and 169 bp for the 5T, 7T and 9T alleles, respectively. Therefore there is no need for fluorescent labeling of the allele-specific primers and the PCR products can be easily distinguishable on agarose gels. However, the allele-specific primers are long and difficult to synthesize. It results in much reduced yield and is therefore more expensive. Another matter of consideration is that the product is no longer available for the US market.

In this report, we developed a simple and easy-to-implement standalone qualitative multiplex AS-PCR assay using standard length primers (less than 50 nucleotides). Our assay can be utilized as a reflex or standalone test for *CFTR* poly-T track analysis.

## Materials & Methods

### DNA samples

Genomic DNA (gDNA) samples from 115 individuals were included in this study. Of these samples, 60 (Table 1) were purchased from Coriell (Coriell Cell Repositories, Camden, New Jersey), including 23 samples from MUTCF-2 Cystic Fibrosis Mutation Panel. The remaining 55 samples (Table S1) were leftover human gDNA from routine clinical tests conducted in Molecular Pathology Laboratory at Beaumont Health System. Of all the samples, only MUTCF-2 Cystic Fibrosis panel had been poly-T track tested previously (Coriell Institute, 2009a; Coriell Institute, 2009b; Sebastian et al., 2004). All the Beaumont samples were de-identified and the study was approved by Human Investigational Committee (HIC 2014-010) at Beaumont Health System.

**Table 1:** Poly-T analysis of Coriell gDNA.

Sample ID	Poly-T alleles	Reference method	Coriell ID^a^	Sample ID	Poly-T alleles	Reference method	Coriell ID^a^
S1	7/7	Sanger sequencing	CD00003	S31	7/7	Sanger sequencing	NA20737
S2	7/9	Sanger sequencing	CD00009	S32	7/7	Sanger sequencing	NA20741
S3	7/7	Sanger sequencing	NA00059	S33	7/7	Sanger sequencing	NA20745
S4	7/7	Sanger sequencing	NA00112	S34	7/7	Sanger sequencing	NA20836
S5	7/7	Sanger sequencing	NA00852	S35	7/7	Sanger sequencing	NA20925
S6	7/9	Sanger sequencing	NA01031	S36	7/9	Sanger sequencing	NA21551
S7	7/7	Sanger sequencing	NA01607	S37	7/9	Sanger sequencing	GM22063^b^
S8	7/7	Sanger sequencing	NA02528	S38	9/9	Other methods^c^	NA01531
S9	7/7	Sanger sequencing	NA02533	S39	7/9	Other methods^c^	NA07441
S10	7/9	Sanger sequencing	NA02828	S40	7/7	Other methods^c^	NA11277
S11	7/7	Sanger sequencing	NA03252	S41	5/7	Other methods^c^	NA11723
S12	7/7	Sanger sequencing	NA03403	S42	5/9	Other methods^c^	NA13591
S13	7/7	Sanger sequencing	NA03441	S96	7/9	Other methods^c^	NA07552
S14	7/7	Sanger sequencing	NA03461	S97	7/9	Other methods^c^	NA08338
S15	7/7	Sanger sequencing	NA04268	S98	7/9	Other methods^c^	NA11275
S16	7/7	Sanger sequencing	NA04394	S99	7/9	Other methods^c^	NA11280
S17	7/7	Sanger sequencing	NA04863	S100	9/9	Other methods^c^	NA11281
S18	7/7	Sanger sequencing	NA07854	S101	7/9	Other methods^c^	NA11282
S19	7/7	Sanger sequencing	NA07857	S102	9/9	Other methods^c^	NA11283
S20	7/7	Sanger sequencing	NA08752	S103	7/9	Other methods^c^	NA11284
S21	7/9	Sanger sequencing	NA09787	S104	7/9	Other methods^c^	NA11472
S22	7/9	Sanger sequencing	NA11285	S105	9/9	Other methods^c^	NA11496
S23	7/7	Sanger sequencing	NA12794	S106	7/7	Other methods^c^	NA11859
S24	7/7	Sanger sequencing	NA13033	S107	7/7	Other methods^c^	NA11860
S25	7/7	Sanger sequencing	NA13205	S108	7/7	Other methods^c^	NA12444
S26	7/7	Sanger sequencing	NA16193	S109	7/7	Other methods^c^	NA12585
S27	7/7	Sanger sequencing	NA17821	S110	7/7	Other methods^c^	NA12785
S28	7/7	Sanger sequencing	NA18802	S111	7/7	Other methods^c^	NA12960
S29	7/9	Sanger sequencing	NA18803	S112	7/9	Other methods^d^	NA18799
S30	7/7	Sanger sequencing	NA18929	S113	7/9	Other methods^e^	NA18800

**Notes.**

a All the gDNA purchased from Coriell were purified by Qiagen Autopure LS instrument, except GM22063.

b GM22063 was purchased as a frozen cell line. gDNA was purified manually by Gentra kit.

c Different methods by different laboratories for poly-T testing were described by Sebastian et al. (2004).

d Poly-T testing was described by Coriell Institute (2009a).

e Poly-T testing was described by Coriell Institute (2009b).

In-house gDNA from sample ID number S43 to S66, S89 and S90 (Table S1) were extracted from peripheral blood using the Gentra Puregene Blood Kit (Gentra Systems, Minneapolis, MN) following the manufacturer’s instructions. The gDNA concentration was measured using a NanoDrop spectrophotometer (Thermo Fisher Scientific, Wilmington, MA). NucliSens easyMAG (bioMerieux, Durham, NC), an automated system for total nucleic acid extraction, was used to isolate the remainder of the in-house gDNA (sample ID number S114 to S142, Table S1). Briefly, red blood cells from 200 µL of EDTA whole blood were lysed by adding 600 µL of RBC Lysis Solution (Qiagen, Germantown, MD). After centrifugation of the lysate, the pellets were resuspended in 1 mL of easyMAG Lysis Buffer (bioMerieux) and transferred to the extraction cassettes for on-board easyMAG extraction, using default Specific A protocol. Then 70 µL of easyMAG elution buffer was added to elute gDNA from the magnetic silica.

### Cloning of 5T alleles

Because there were no samples from the MUTCF-2 Cystic Fibrosis Mutation Panel that are homozygous for 5T alleles, we cloned 5T alleles from NA11723 and NA13591, two of samples from MUTCF-2 panel. A 267bp fragment was amplified using AmliTaq 360 DNA polymerase (Life Technologies, Carlsbad, CA) and 250 nmol/L of each of the CFTR_ex9_P1 (5′-AAAACAAGCATCTATTGAAAATATCTGA-3′) and CFTR_ex9_P2 (5′-CTTGCCTGCTCCAGTGGAT-3′) primers. PCR products of each sample were cloned into pCRII-TOPO vector (Life Technologies, Carlsbad, CA). Three individual clones from each of the samples, NA11723 and NA13591, were sequenced (ABI 3730*xl* instrument; Elim Biopharmaceuticals, Hayward, CA). Sequencing results indicated that NA11723 has 5T and 7T alleles with 12 and 10 TG repeats, respectively. NA13591 has 5T with 12 TG repeats and 9T with 10 TG repeats. Both 5T alleles contain the same number of TG repeats and would generate the same size AS-PCR products. A 5T plasmid clone from NA13591 was used in the study.

### Primer design and multiplex AS-PCR

**Figure 1:**
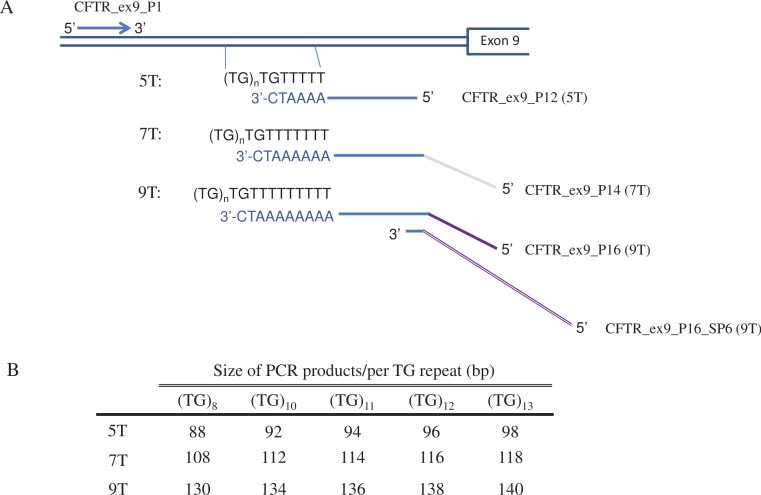
Primer design for *CFTR* poly-T analysis. (A) Diagram of the AS-PCR primers. The multiplex AS-PCR has a common upstream primer, CFTR_ex9_P1, and allele specific downstream primers, CFTR_ex9_P12 , CFTR_ex9_P14, and CFTR_ex9_P16 for 5T, 7T and 9T alleles, respectively. In order to distinguish the 5T, 7T and 9T by the size of the final AS-PCR products, a third primer, CFTR_ex9_P16_SP6, was used in the multiplex PCR reaction for 9T alleles. (B) Size of the AS-PCR products for 5T, 7T, and 9T alleles. Considering TG repeats could range from 8 to 13, PCR products for 5T, 7T and 9T could be from 88 to 98bp, 108 to 118bp, and 130 to 140bp, respectively.

Three sets of primers were designed to identify 5T, 7T or 9T allele in the intron 8 of *CFTR* (Fig. 1). All have the same upstream primer CFTR_ex9_P1 (5′-AAAACAAGCATCTATTGAAAATATCTGA-3′), but three different downstream primers, CFTR_ex9_P12 (5′-TTCCCCAAATCCCTGTTAAAATC-3′) for 5T, CFTR_ex9_P14 (5′-CGTTGTAAAACGACGGCCAGCCCCAAATCCCTGTTAAAAAATC-3′) for 7T, and CFTR_ex9_P16 (5′-ACCTAAATAGCTAAGCCCAAATCCCTGTTAAAAAAA ATC-3′) and CFTR_ex9_P16_SP6 (5′-GCATAGCTTGAGTATTCTATAGTGTCACCTAAATAGCTAAGC CCAAATC-3′) for 9T. PCR product size for each allele is different, 88 to 98 bp for 5T, 108 to 118 bp for 7T and 130 to 140 bp for 9T, considering TG repeats at 5′ of the poly-T track range from 8 to 13.

The multiplex AS-PCR is carried out in a 10-µL reaction containing 1X Melt Doctor HRM master mix (Applied Biosystems, Foster City, CA), 25 ng of the Coriell, in-house manually extracted or 1 µL of the easyMAG extracted gDNA, and 750 nmol/L of CFTR_ex9_P1, 250 nmol/L of CFTR_ex9_P12 and CFTR_ex9_P16_SP6, 100 nmol/L of CFTR_ex9_P14 and 50 nmol/L of CFTR_ex9_P16. PCR was performed at 95 °C for 5 min, followed by 36 cycles at 95 °C for 15 s, 59 °C for 30 s and 72 °C for 45 s. An additional 72 °C for 5 min extension was added at the end of the 36-cycle reaction. PCR products were separated by size using the Qiaxcel DNA High Resolution cartridge and method OM800 on the Qiaxcel Advanced System (Qiagen) to determine whether samples contain 5T, 7T or 9T allele or a combination of any two of the alleles. For 5T plasmid, 170 pg of plasmid were added to the multiplex AS-PCR.

### Evaluation of run-to-run and within-run reproducibility of the multiplex AS-PCR

After optimization of the multiplex AS-PCR, run-to-run and within-run reproducibility were evaluated using five gDNAs from MUTCF-2 panel, NA01531 (9/9T), NA07441 (7/9T), NA11277 (7/7T), NA11723 (5/7T) and NA13591 (5/9T), and a 5T plasmid clone as homozygous 5/5T. The final call of the poly-T alleles of each sample was compared to determine the reproducibility of the method. For 5T plasmid, 170 pg of plasmid were added to the multiplex AS-PCR.

### Poly-T analysis using multiplex AS-PCR and confirmed with sanger sequencing

110 samples were tested to determine the length of the poly-T alleles using the newly developed multiplex AS-PCR. Among these samples, eighteen (sample ID S96 to S113) from MUTCF-2 of Coriell Cell Repositories were tested previously (Coriell Institute, 2009a; Coriell Institute, 2009b; Sebastian et al., 2004). Poly-T track of the remaining 92 samples (sample ID S1 to S37, S43 to S66, S89, S90 and S114 to S142) were unknown. Multiplex AS-PCR was set up the same way as in “Primer Design and Multiplex AS-PCR”.

Sequences flanking the poly-T region of all the 92 samples of unknown poly-T alleles were amplified using primers CFTR_ex9_P1 and CFTR_ex9_P2 as in “Cloning of 5T Alleles”. Each PCR reaction contained 1X Melt Doctor HRM master mix, 250 nmol/ul of each of the primers and 50 ng of Coriell or in-house manually extracted or 2 µL of the easyMAG extracted gDNA. PCR products were run on Qiaxel to determine the proper amplification of a single 267 bp fragment. Then PCR products were treated with Exonuclease and Shrimp Alkaline Phosphatase based on manufacturer’s instruction (Affymetrix, Santa Clara, CA). CFTR_ex9_P2 were used for Sanger sequencing (Elim Biopharmaceutical). Poly-T status of each sample was determined from directly reading the sequencing chromatogram.

## Results

### Primer design and optimization of the multiplex AS-PCR reactions

Each PCR primer set was designed to uniquely amplify 5T, 7T or 9T in intron 8 of *CFTR* (Fig. 1A). The downstream primers are complementary to the corresponding length of poly-T sequences, which confers the specificity for each poly-T allele. The primers end with a 3′-C that is complimentary to the G in the last TG repeat of the template sequence. To increase the specificity of the allele-specific primer binding, an additional deliberate mismatch (i.e., from A to T) at the next to the last base of the allele-specific primers was introduced (Little, 2001; Wangkumhang et al., 2007).

A single multiplex AS-PCR was intended to detect a sample with the possibility of being homozygous for any one of poly-T alleles or heterozygous for any two combinations, 5T/7T, 7T/9T or 5T/9T. As fluorescent labeled primers are expensive and need special instrument for fragment analysis, unlabeled primers that can generate fragments in different sizes would be ideal for wider adaption of the assay (Bayliffe et al., 2001; Elucigene Diagnostics, 2004). For this reason, the overhang DNA sequences were added to the 5′ end of the AS-PCR primers to generate PCR products with at least 10 bp difference in size among all three poly-T alleles (Figs. 1A and 1B). These PCR products are then easily distinguishable on Qiaxcel DNA High Resolution cartridge.

AS-PCR products for the same poly-T allele, such as 5T, can vary up to 10 bp in size because each poly-T allele may contain 8 to 13 TG repeats at the 5′ end of the poly-T tract (Fig. 1B). Primer CFTR_ex9_P12 for 5T consists of only *CFTR* sequences to generate the shortest PCR amplicon (88 bp to 98 bp). CFTR_ex9_P14, a 43-base primer for 7T includes 20 bases of M13 sequences added to the 5′ of the primer, generating fragment size of 108 to 118 bp. A 61-base or longer AS-PCR primer would be designed to generate the shortest 9T allele (i.e., a minimum 128 bp PCR product with 8 TG repeats), that is at least 10-bp longer than the largest 7T allele (7T with 13 TG repeats, which is 118 bp). Technically, synthesis of such long primers is possible. The longest primer for Elucigene’s AS-PCR is 90 bases to detect 9T alleles (Iain et al., 2001). However, there are challenges in quality and consistency, as well as in purification of the complete primers from truncated premature products (Kozlov et al., 2005). To prevent synthesizing a long oligo for the 9T allele, we have used two-downstream-primer approach for 9T amplification. CFTR_ex9_P16, a 39-base primer, contains 24 bases of CFTR complementary sequence and a 15-base 5′ overhang of SP6 promoter. CFTR_ex9_P16_SP6, a 49-base primer, has a 23-base overlap with 5′ of CFTR_ex9_P16 and 26-base of additional SP6 promoter sequence at its 5′ end. The total length of the PCR products for 9T is between 130 and 140 bp. It allows detection of each poly-T allele using High Resolution Qiaxcel cartridge, without labeling of the downstream primers.

**Figure 2:**
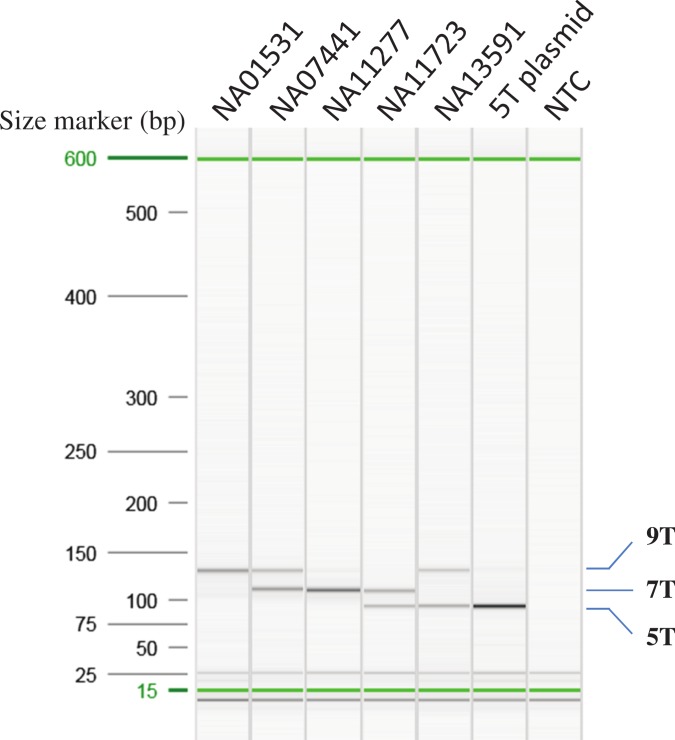
Optimization of AS-PCR for poly-T analysis. All the samples used for optimization of the multiplex AS-PCR have known poly-T allele information: NA01531 (9/9T), NA07441 (7/9T), NA11277 (7/7T), NA11723 (5/7T) and NA13591 (5/9T). 5T plasmid was cloned from NA11723 and NA13591. After Sanger sequencing of the plasmid clones, the 5T allele has 12 TG repeats, the 7T allele from NA11723 has 10 TG repeats, and the 9T allele from NA13591 has 10 TG repeats. All the AS-PCR products were run on the High Resolution cartridge of Qiaxcel Advanced System. NTC: No template control.

Different combinations of the primer concentration and annealing temperature were tested to optimize the multiplex AS-PCR. Figure 2 indicated that the optimized multiplex AS-PCR is able to detect all six combinations of the poly-T alleles in samples of known poly-T length from the MUCF-2 panel: NA01531 (9/9T), NA07441 (7/9T), NA11277 (7/7T), NA11723 (5/7T) and NA13591 (5/9T), and a cloned 5T allele as 5/5T. Furthermore, we also tested the remaining 18 samples from MUTCF-2 panel; all matched to the published data for the poly-T status (Table 1).

### Run-to-run and within-run reproducibility

The same five samples and 5T plasmid used for optimization of the multiplex AS-PCR reaction were tested for within-run reproducibility (run in triplicate on the same PCR plate) and for run-to-run reproducibility (three different runs on the same PCR instrument). All indicated that the multiplex AS-PCR was able to determine the poly-T alleles for all six poly-T allele combinations in the same run or different runs (data not shown).

### Poly-T analysis of unknown samples

**Figure 3:**
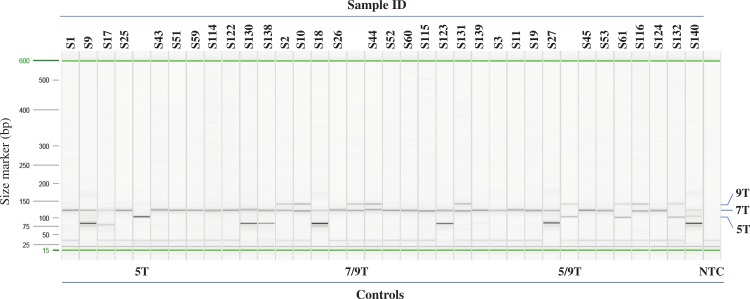
Example of Poly-T analysis using multiplex AS-PCR. Results of AS-PCR were listed in Table 1 and Table S1 and confirmed by published data or Sanger sequencing. Sample ID from S1 to S27 are from Coriell. Sample ID from S43 to S61 and S114 to S140 are from Beaumont Health System. Control 5T, 7/9T and 5/9T are cloned plasmid DNA, NA07441 and NA13591, respectively. NTC: no template control.

**Figure 4:**
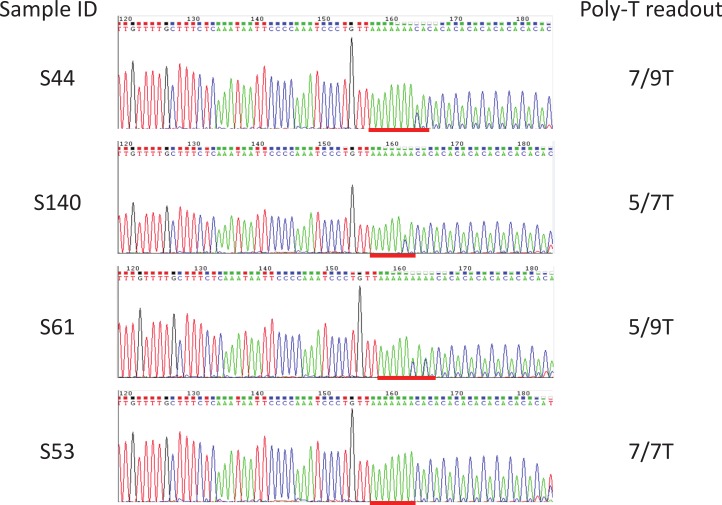
Examples of sequencing results for unknown samples. Sanger sequencing was used as a reference method to determine the poly-T status of all the unknown samples. Included were Coriell gDNA of samples S1–S37, and in-house gDNA of samples S43–S66, S89, S90, and S114 to S142. All the poly-T allele results were listed in Table 1, Fig. S1. Poly-T sequences were highlighted with red lines.

92 samples with unknown poly-T status were tested using the multiplex AS-PCR and run on Qiaxcel DNA High Resolution cartridge (Fig. 3, Table 1 and Table S1). After AS-PCR testing, poly-T tracks of all 92 samples were confirmed by Sanger sequencing (Fig. 4). Results for poly-T allele status from multiplex AS-PCR matched results from Sanger sequencing for all samples.

## Discussion

We developed a multiplex AS-PCR to simultaneously detect 5T, 7T and 9T in the poly-T region of intron 8 of *CFTR*. Our assay was able to detect all the combinations of the poly-T alleles, 5/5T, 7/7T, 9/9T, 5/7T, 5/9T, and 7/9T (Fig. 2). Out of the 115 human gDNA samples tested, only the MUTCF-23 panel had known poly-T results. The rest of the samples were Sanger sequenced to determine the poly-T length. All the results from multiplex AS-PCR matched to the published or Sanger sequencing results, indicating that our assay had 100% sensitivity in detecting all types of poly-T alleles and 100% specificity to correctly detect poly-T alleles in patient samples. Thus, it is a reliable method for poly-T analysis.

The main advantage of our assay is its simplicity which is a relevant criterion for easy adaptation in clinical and research laboratories. One improvement over the Elucigene’s single tube multiplex AS-PCR is to split the longest AS-PCR primer to two shorter oligos that can be synthesized without special care and purification overhead required with longer oligos.

However, there are a few considerations to establish the assay in a laboratory. First, annealing temperature is critical as allele-specific amplification is based on the difference of the annealing temperature of each allele-specific primer to generate the corresponding PCR products among the alleles, thus annealing temperature must be tested on each instrument used for this assay. It is especially sensitive in this assay because the 5T, 7T and 9T alleles differ in two or four bases of the thymidine repeats. We have found that programming the same annealing temperature on a different PCR instrument didn’t work as well as the initial one used for optimization. Once we slightly adjusted annealing temperature (i.e., higher or lower than the optimized annealing temperature on our first testing instrument) on that instrument, we were able to obtain consistent results for poly-T testing. The main objective is to calibrate the annealing temperature on each instrument so that the specific target products are maximally amplified, whereas the non-specific by-products are minimized.

Second, using a PCR instrument with good uniformity across all the wells would produce better reproducibility and more robust results. Most of PCR instruments are block-based and temperatures are known to vary among different wells of the same block (Logan & Edwards, 2009; Saunders et al., 2001). Such variations would lead to inconsistency since the actual annealing temperature of the AS-PCR in each individual well may not be identical. We observed that when the variation of the temperature exceeds the optimal annealing temperature to generate allele-specific PCR products, the reaction would fail, i.e., no products or non-specific PCR products were generated. Testing a group of samples with known poly-T status, such as MUTCF-2 panel, would verify whether the uniformity of the PCR block is sufficient to produce robust and correct results. Third, we used an automated high resolution gel cartridge of Qiaxcel Advanced System to detect the size of the AS-PCR products. However, with at least 10 bp difference in each of AS-PCR products, running the multiplex AS-PCR products on high concentration agarose gel worked equally well (Fig. S1).

Lastly, interpretation of the poly-T results needs to take the size range of each allele into consideration (Fig. 1B). With the variable number of TG repeats within the AS-PCR products, the same poly-T allele, such as 5T, could range from 88 bp to 98 bp (Fig. 1B). That is, the same 5T allele can generate two bands with 10 bp difference in size. However, a sample with two different poly-T alleles could generate two bands with 10 bp different in size, such as 5T with 13 TG repeats and 7T with 8 TG repeats. To determine whether two bands are two different alleles or the same poly-T allele, the size range of each allele is the key to make the correct call (Fig. 1B). Each allele corresponds with an individual size range, which does not overlap among different alleles. We recommend running control samples with known PCR product size, such as NA11723 and/or NA13591with 5T, 7T and 9T, along with unknown testing samples to facilitate data interpretation. In all our test samples, we didn’t see any samples with visible size difference for the same allele. This may be due to the fact that the most common TG repeats are 11, 12 and 13 (Moskowitz et al., 2008). Therefore the most common scenario would be the same allele with at most 6 bp difference in length (i.e., 3 TG repeats from 11 to 13), and different alleles with at least 16 bp difference in length, such as 5T plus 13 TG repeat vs 7T with 11TG repeat (Fig. 1B). It would be relatively easy to distinguish the same two poly-T from different two poly-T alleles .

Although the assay performed well in our hands, each lab would need to establish the protocol on its own instrument to implement the test. The first step would be to choose the PCR instrument with the best temperature uniformity in a laboratory. Instruments with multiple temperature sensors usually have better uniformity than a single sensor. Secondly, five of the 23 samples from MUTCF-23 panel with different poly-T alleles (Fig. 2) would be used to adjust the annealing temperature on the instrument. Subsequently, additional samples with known poly-T alleles, such as the MUTCF-23 panel, could be tested to determine whether the multiplex AS-PCR generates correct results. Optimal uniformity of the thermal block is indicated by 100% concordance with the known results; any discordance of results indicates uneven temperature of the thermal block, if everything else is done correctly. The lab may need to seek advice from the instrument vendor to evaluate the temperature variability of the instrument. With moderate volume of poly-T test in a lab, the thermal block can be virtually partitioned; that is, only a portion of the block with good temperature uniformity will be used to validate and perform the assay. Then, run-to-run and within-run reproducibility can be performed to assess the robustness of the assay. The last step would be to perform a blind test of more samples to establish the analytical specificity of the assay. If samples are tested with unknown poly-T allele status, Sanger sequencing, a gold standard for variant detection, can be implemented to confirm the AS-PCR results (Fig. 4).

Overall, we developed a simple, easy to implement standalone multiplex AS-PCR method to detect all three poly-T alleles in intron 8 of *CFTR*. Our assay can be implemented as a reflex test in patients with positive R117H, if another method for *CFTR* mutation analysis didn’t include poly-T analysis. The assay can also be combined with any routine *CFTR* tests as a standalone method for molecular diagnosis of CBAVD. Furthermore, our AS-PCR design improves robustness and consistency for poly-T allele detection in *CFTR*.

## Supplemental Information

10.7717/peerj.468/supp-1 Figure S1 Agarose gel electrophoresis of the AS-PCR for poly-T analysis All the samples used for optimization of the multiplex AS-PCR have known poly-T allele information, NA01531 (9/9T), NA07441 (7/9T), NA11277 (7/7T), NA11723 (5/7T) and NA13591 (5/9T). 5T plasmid was cloned from NA11723 and NA13591. After Sanger sequencing of the plasmid clones, all the clones with 5T has 12 TG repeats, 7T allele from NA11723 has 10 TG repeats, and 9T allele from NA13591 has 10 TG repeats. 5 µl of the AS-PCR reactions were run on 4% NuSieve 3:1 agarose gel (Lonza, Rockland, ME). NTC: No template control.

10.7717/peerj.468/supp-2 Table S1 Poly-T analysis of in-house gDNA
