# Supplementary material for: Development of allele-specific multiplex PCR to determine the length of poly-T in intron 8 of CFTR
Source: PeerJ. 2014 Jul 8;2:e468. doi: 10.7717/peerj.468 (PMC4103085; doi:10.7717/peerj.468)
Supplement: Table S1 [file peerj-02-468-s002.docx]

Supplemental Table 1. Poly-T analysis of in-house gDNA.

| **Sample ID** | **Poly-T alleles^1^** | | **Extraction method** | |  | | **Sample ID** | | **Poly-T alleles^1^** | **Extraction method** | |  |  |
| --- | --- | --- | --- | --- | --- | --- | --- | --- | --- | --- | --- | --- | --- |
| S43 | | 7/7 | | Gentra kit | |  | | S116 | 7/9 | EasyMag | | | |
| S44 | | 7/9 | | Gentra kit | |  | | S117 | 7/7 | | EasyMag | |  |
| S45 | | 7/7 | | Gentra kit | |  | | S118 | 7/9 | | EasyMag | |  |
| S46 | | 7/9 | | Gentra kit | |  | | S119 | 7/7 | | EasyMag | |  |
| S47 | | 7/7 | | Gentra kit | |  | | S120 | 7/7 | | EasyMag | |  |
| S48 | | 7/7 | | Gentra kit | |  | | S121 | 7/7 | | EasyMag | |  |
| S49 | | 7/7 | | Gentra kit | |  | | S122 | 7/7 | | EasyMag | |  |
| S50 | | 7/7 | | Gentra kit | |  | | S123 | 7/7 | | EasyMag | |  |
| S51 | | 7/7 | | Gentra kit | |  | | S124 | 7/7 | | EasyMag | |  |
| S52 | | 7/7 | | Gentra kit | |  | | S125 | 7/9 | | EasyMag | |  |
| S53 | | 7/7 | | Gentra kit | |  | | S126 | 7/7 | | EasyMag | |  |
| S54 | | 7/7 | | Gentra kit | |  | | S127 | 7/9 | | EasyMag | |  |
| S55 | | 7/7 | | Gentra kit | |  | | S128 | 7/7 | | EasyMag | |  |
| S56 | | 7/7 | | Gentra kit | |  | | S129 | 7/7 | | EasyMag | |  |
| S57 | | 7/7 | | Gentra kit | |  | | S130 | 7/7 | | EasyMag | |  |
| S58 | | 7/7 | | Gentra kit | |  | | S131 | 7/9 | | EasyMag | |  |
| S59 | | 7/7 | | Gentra kit | |  | | S132 | 5/9 | | EasyMag | |  |
| S60 | | 7/7 | | Gentra kit | |  | | S133 | 7/7 | | EasyMag | |  |
| S61 | | 5/9 | | Gentra kit | |  | | S134 | 7/7 | | EasyMag | |  |
| S62 | | 5/9 | | Gentra kit | |  | | S135 | 7/7 | | EasyMag | |  |
| S63 | | 5/7 | | Gentra kit | |  | | S136 | 7/9 | | EasyMag | |  |
| S64 | | 7/7 | | Gentra kit | |  | | S137 | 7/9 | | EasyMag | |  |
| S65 | | 7/9 | | Gentra kit | |  | | S138 | 7/7 | | EasyMag | |  |
| S66 | | 7/9 | | Gentra kit | |  | | S139 | 7/7 | | EasyMag | |  |
| S89 | | 7/7 | | Gentra kit | |  | | S140 | 5/7 | | EasyMag | |  |
| S90 | | 7/7 | | Gentra kit | |  | | S141 | 7/9 | | EasyMag | |  |
| S114 | | 7/7 | | EasyMag | |  | | S142 | 7/9 | | EasyMag | |  |
| S115 | | 7/7 | | EasyMag | |  | |  |  | |  | |  |

^1^All the results are confirmed by Sanger sequencing.
